# Annotations

**Published:** 1911-01-07

**Authors:** 


### ANNOTATIONS.

### "Grinding" in Ireland.

IT is announced that the Council of the Royal College of Surgeons of Ireland has decided on certain regulations designed to hamper the operations of the private surgical examination coach, commonly known in Irish schools as the "grinder." In future none but Fellows of the College are to be allowed to undertake private pupils, and even these must be duly nominated by the Dean of their school and approved by the Council. Every such licensed teacher must pay an annual fee of five guineas and a capitation sum for every pupil he undertakes. This latter payment has been fixed at a price which will practically extinguish coaching, for it is said to be in itself about as much as the ordinary fee which such coaches charge. In so far as it is designed to suppress entirely the evil of cramming for surgical examinations, the regulation is perhaps one to be applauded; but it is to be feared that one of two things will happen—either the law will be ingeniously evaded or else the Royal College will lose so large a proportion of students that it will eventually be relaxed. We hold no brief for "grinding"; on the contrary, we regard it as unnecessary and demoralising, the former because the school teaching staff and the excellent text-books on every surgical subject should be quite enough for any diligent student, even if his wits be not over sharp, and the latter because the machine-crammed student is in most cases he who has neglected his ward studies and is little likely to advance the honour and reputation of the profession when he gets into practice. Yet we believe that restrictive measures such as these are unwise and tend to defeat their own purpose. It should be the function of the College's examiners to detect and reject him whose knowledge of surgery is merely superficial and got by eleventh-hour coaching, and the examinations should distinguish from such students those whose knowledge has been acquired by diligent clinical study.

### Motherliness.

THE National League for Physical Education and Improvement does a great deal of good in a quiet and unobtrusive way. Its educative work is carried on mainly by the distribution of excellently compiled pamphlets which give a mass of valuable information in a commendably simple and lucid manner. One of these pamphlets is the report on existing "Schools for Mothers" which has been compiled by I. G. Gibbon; it deals with those institutions where parents are advised with regard to the rearing of children, and where the mothers are instructed on the simple but so vitally important points of infant hygiene. There are already nearly a hundred such institutions in active co-operation with various benevolent and health associations, and the number is increasing while the institutions themselves are rapidly becoming more popular. The fact that they are on the increase is at once a proof of their usefulness and a sign of the necessity for persevering with the instruction of motherliness. The community, especially in overcrowded districts, derives a vast amount of benefit from systematic health work of this nature; the force of example, even more than that of precept, is nowhere so conspicuous as where it concerns the prevention of infantile mortality and infantile ill-health. We hear a great deal about the first, but the second is almost equally important from a national point of view, and it is encouraging to find that the Mothers' Schools are so actively supported.

### The New Year Honours.

THE list of honours conferred by his Majesty the King contains an unusual number of distinctions for officials and politicians in the Colonies, a natural tribute to the growing importance of these bulwarks of our Empire. As far as the medical profession is concerned, the most noteworthy of these honours is the baronetcy conferred upon the celebrated "Dr. Jim," L. S. Jameson, M.D., C.B., physician, administrator, raider, soldier, and politician. In the same list appears the name of Senator J. H. M. Beck, M.D., who becomes K.C.B. In our great Eastern Dependency Lieut.-Colonel David Semple, M.D., Royal Army Medical Corps, becomes a knight bachelor for research work at Kasauli; and the Kaisar-i-Hind medal is conferred upon Lieut.-Colonel Henry Smith, M.D., I.M.S.; Major T. W. Irvine, M.B., I.M.S.; and Mrs. Edwin Davies, Chief Lady Superintendent of Lady Minto's Indian Nursing Association. Turning to the British Islands, Dr. H. B. Donkin, an old St. Thomas's man, well-known for his activities as medical adviser to the Prison Commissioners and for his contributions to the study of heredity and crime, becomes a knight. He is consulting physician to the Westminster Hospital, the East London Hospital for Children, and the New Hospital for Women; and examiner in medicine for the Royal College of Physicians. A like honour, and equally well deserved, falls to Dr. David Ferrier, Mr. F. S. Eve, and Dr. George Newman. The first has been for many years one of the premier neurologists of the world; he is consulting physician to King's College Hospital and to the National Hospital for the Paralysed and Epileptic. Dr. Ferrier was educated at Edinburgh University. The second is almost as well known; he is senior surgeon to the London and the Evelina Hospitals, but was originally a Bart.'s man. The last-named is chief medical officer to the Board of Education, and is recognised as one of the most capable and energetic officers the public medical service has ever had. He also is an Edinburgh man. The remaining medical knighthood goes to Ireland, in the person of Mr. J. M. Redmond, who was President of the Royal College of Physicians of Ireland in 1906-08. He is M.D. Brussels, and physician to the Mater Misericordiæ Hospital, Dublin; also consulting physician to the Coombe Hospital, Dublin, and to St. Michael's Hospital, Kingstown.

